# Intercontinental translatability of a peer-led GP education model from New Zealand to the UK: a pilot study

**DOI:** 10.3399/BJGPO.2023.0227

**Published:** 2024-08-21

**Authors:** Ben Hudson, Matthew Walmsley, Susan Bidwell, Louise Kennedy

**Affiliations:** 1 Department of Primary Care and Clinical Simulation, University of Otago, Christchurch, New Zealand; 2 Lyttelton Health Centre, Christchurch, New Zealand; 3 Pegasus Health, Christchurch, New Zealand; 4 Marsden Medical Group, South Shields, UK; 5 South Tyneside & Sunderland NHS Foundation Trust, Tyne and Wear, UK; 6 Clinical Quality and Education, Pegasus Health, Christchurch, New Zealand

**Keywords:** continuing professional development, general practitioners, New Zealand, pilot projects, primary health care, United Kingdom, videoconferencing

## Abstract

**Background:**

Pegasus Small Group education for GPs is a professional development programme that has been delivered in Canterbury, New Zealand for over 30 years. Peer-developed content is delivered in small groups supporting interactive discussions informed by evidence and locally relevant data.

**Aim:**

An international collaboration between South Tyneside Clinical Commissioning Group in the UK and Pegasus Health in Canterbury, New Zealand aimed to determine whether the Canterbury model of Small Group professional development for GPs was transferrable to the South Tyneside context.

**Design & setting:**

This was a pilot qualitative study testing proof of concept for the Pegasus Small Group GP education model of professional development in another country.

**Method:**

To test the concept, three pilot sessions on persistent pain, screening, and optimising treatment were delivered between November 2021 and March 2022. Four UK GPs were trained as Small Group leaders, and a member of the Pegasus team liaised with various UK GPs in South Tyneside to adapt topics for the local context. The use of videoconferencing (Microsoft Teams and Zoom) to deliver support, training, and the programme itself had been developed and refined during the COVID-19 pandemic, so that it could be run entirely online without losing its core components or interactive nature.

**Results:**

Of the 68 registered GPs, 31, 50, and 61 GPs attended the three sessions, respectively, 90% of whom rated the overall quality as good or excellent. These results and other positive feedback from attendees provided a mandate for a further extension of the programme over the following months.

**Conclusion:**

The pilot proved the potential for health systems to collaborate globally despite geographical distance. A wider evaluation to assess the impact of the education initiative is needed to determine the impact on patient care and to demonstrate the benefits of supporting the small group peer education model.

## How this fits in

The Pegasus Small Group peer education model for GP professional development has been delivered across different regions of New Zealand for many years. This groundbreaking pilot outlines the international delivery of the model for the first time in South Tyneside, UK. The results show the acceptability and translatability of this professional development model for GPs in South Tyneside and, more broadly, highlight the possibilities and potential efficiencies achieved through global collaborations.

## Introduction

In 2021 an international collaboration between the South Tyneside Clinical Commissioning Group (CCG) in the UK and Pegasus Health in Canterbury, New Zealand aimed to test whether the Pegasus Small Group model of professional development used in Canterbury could be successfully transferred to the South Tyneside context. The South Tyneside CCG covers 21 general practices with approximately 70 practising GPs. The organisation supports GP professional development by providing release time on one Thursday a month. This had traditionally been in a large group lecture-style format.

The CCG was looking for change, and was aware of evidence that while didactic methods could be effective in increasing knowledge, greater knowledge alone was not optimal for improving clinical performance^1–3^ The use of multiple methods and a focus on outcomes important to the participants were more likely to have a greater effect on physician performance, and therefore, patient outcomes.^4^ Interactive group learning, moreover, had proved to be well liked by GPs elsewhere, allowing them to learn from one another’s strengths and experiences and support strategies to implement new knowledge into practice^5,6^ as well as reducing professional isolation and stress and improving practitioner morale.^7^

The CCG was aware of the Pegasus Small Group interactive model of professional development that had been running successfully for almost 30 years in Canterbury, New Zealand.^8^ Rather than attempt to develop their own programme from scratch, they decided that it would be more efficient to adopt an educational approach that was already well proven. They therefore approached Pegasus Health to suggest they work together to test whether the Small Group programme would be transferable to the South Tyneside context as a means of improving the local provision of professional development.^8^

Adopting an international model was not new to the CCG, as they had already successfully collaborated with Streamliners, also based in Canterbury, New Zealand, to implement HealthPathways, an online referral pathways tool that supports primary care clinicians. Pegasus also had previously collaborated with partner primary care groups to offer the programme in other regions of New Zealand .

The Pegasus Small Group programme is based on best practice in adult learning,^9,10^ is peer-developed, and is intentionally delivered in small groups through interactive discussions informed by evidence and local data. Supporting practitioners to deliver high quality patient care, it promotes best practice and supports change through optimal and ethical use of limited health system resources. It is continually reviewed and refined to meet the evolving needs of primary care health professionals.

The collaboration between the groups was enabled using video conferencing (Microsoft Teams and Zoom) to deliver the support and training required. These approaches had been developed, refined, and embedded by the Pegasus team during the COVID-19 pandemic in 2020 using evidence-based principles.^11–13^ This article outlines the process by which a three-topic pilot of the Small Group professional development programme was developed and implemented among the GPs of South Tyneside, and evaluated for transferability.

## Method

Embarking on an international collaboration was an ambitious undertaking both for the CCG and Pegasus Health, even given the evolution of the Pegasus Small Group programme so that it could be delivered equally well in person or remotely to local or distant groups.^14,15^ The New Zealand health system is similar to UK in many respects and has many UK-trained GPs practising there who understand both contexts; nevertheless, the programme required work to adapt it to the UK context.

The CCG leaders worked with the Pegasus team to choose an initial topic and adapt the original New Zealand version so that it was localised to the UK context and aligned with UK clinical pathways. The topic (persistent pain) was presented in September 2021 to clinical leaders within the CCG by lead clinicians from the Pegasus group as a showcase demonstration. It was presented online in real time via Zoom to a group of GPs identified by South Tyneside as potential peer Small Group leaders who could deliver the pilot programme. Following the showcase, an agreement was signed between the two groups to proceed with a pilot of three topics to be presented at the time of the Thursday learning and development sessions in alternate months over the next six months, replacing the usual lecture format in those months. The three topics chosen for the pilot were persistent pain, screening, and optimising treatment. The pilot aimed to achieve similar uptake in attendance rates and evaluation scores to those in New Zealand.

Four volunteer GPs from the CCG group were trained as Small Group leaders via Zoom by the Pegasus lead trainer. Training covered facilitation skills, online etiquette, and ensuring that sessions were interactive and included all participants. An experienced clinical facilitator from the Pegasus team was appointed to liaise with the respective CCG lead in adapting the topics for the South Tyneside context. Local primary care specialists were used to provide additional expert advice. The Education Coordinator for the CCG ran a website promotion to inform potential participants about the Small Group meetings and give them the option to sign up. Three Small Groups were formed around the three primary care networks in the CCG.

An online leaders’ briefing for topic 1 (persistent pain) was held in November 2021, followed by the first Small Group meetings the following week. The two other pilot topics ran in the same way during February 2022 (topic 2, screening) and March 2022 (topic 3, optimising treatment). Small Groups ran concurrently to fit with the release time provided for the South Tyneside clinicians on a Thursday. They met either in person or online, depending on local circumstances, the model having been deliberately developed so that it could be run in either format without losing the core components or interactive nature.^14^ An online catch-up evening meeting was also presented for those who had been unable to attend at the usual time. After each meeting attendees were given 2 weeks to evaluate the session by submitting their ratings in an online form (see Supplementary material). The form, the same as that used by the New Zealand groups, covered four categories: quality of information, relevance of the content, the facilitation skills of the leader, and the overall quality of the meeting. Each category could be rated as poor, average, good, or excellent. The form also prompted attendees to suggest improvements, put forward topics they would like addressed in future, and to add any other comments.

## Results

The three pilot sessions were attended by 31, 50, and 61 GPs, respectively, from a total pool of 68 GPs who had registered to participate. Sixteen, 18, and 18 completed evaluations were available for analysis from the first, second, and third topics, respectively. Results are shown in Table 1.

**Table 1.** South Tyneside participant evaluation scores for pilot topics

		Persistent pain 15 evaluations, *n/N* (%)	Screening 18 evaluations *n/N* (%)	Optimising treatment 18 evaluations, *n/N* (%)
**Quality of information**	Average	2/15 (13.3)	2/18 (11.1)	3/18 (16.7)
	Good	7/15 (46.7)	9/18 (50.0)	7/18 (38.9)
	Excellent	6/15 (40.0)	7/18 (38.9)	8/18 (44.4)
**Relevance**	Average	2/15 (13.3)	1/18 (5.6)	1/18 (5.6)
	Good	8/15 (53.3)	11/18 (61.1)	9/18 (50.0)
	Excellent	5/15 (33.3)	6/18 (33.3)	8/18 (44.4)
**Facilitation**	Average	1/15 (6.7)	2/18 (11.1)	1/18 (5.6)
	Good	9/15 (60.0)	9/18 (50.0)	5/18 (27.8)
	Excellent	5/15 (33.3)	7/18 (38.9)	12/18 (66.7)
**Overall quality**	Average	1/15 (6.7)	2/18 (11.1)	1/18 (5.6)
	Good	11/15 (73.3)	10/18 (55.6)	9/18 (50.0)
	Excellent	3/15 (20.0)	6/18 (33.3)	8/18 (44.4)

As Table 1 shows, 90% of the evaluations for the first and third topics rated their overall quality as good or excellent, with the screening topic slightly behind at around 89%. A rating of ‘poor’ was also available, but no ‘poor’ ratings were given for any of the three meetings in any category. Comments from participants were also positive for all three topics:

‘*Brilliant session, very holistic and maximised my knowledge and dealing with clinical presentations and pain management*.’ (Topic 1)

‘*Good information re*[garding] *screening and managing non-responders*.’ (Topic 2)

‘*Very relevant to general practice; application of data and information change behaviour of prescribing, decision sharing*.’ (Topic 3)

The mix of presentation and interactive discussion proved popular, with many positive comments on the benefits of learning from the experience of other GPs:

‘*Excellent session, well run, good fun, engaging, interesting and relevant. Good mix between discussion and presentation*.’ (Topic 2)

Participants also noted changes to their clinical practice that they intended to make after attending the meetings, including encouraging shared decision making, using the prescribing tools and resources highlighted in the presentation, and liaising with other health professionals.

The meeting on screening was less highly rated than the other two, being rated as average by two of 18 responders across three of the four categories. Comments noted it was ‘*too long, no new information, and a bit dry’*. However, others commented that it was ‘*very relevant, useful discussion, good information re*[garding] *managing non-responders’*. There were some concerns that the data and terminology were not sufficiently relevant to the local context, but these resolved over the course of the pilot.

Suggested improvements related mainly to the sound quality of the online briefings and meetings, and facilitators who failed to address non-engagement by some participants who had their cameras off. These too seemed to resolve as the pilot progressed:

‘*I loved the way* [the facilitator] *asked the quieter people and tried to include them. Think having the cameras on made a big difference*.’ (Topic 3)

Overall comments on all three of the meetings were overwhelmingly positive:

‘*Excellent session, lots of food for thought on wordings used. Lots of resources to signpost patients to*.’ (Topic 1)

‘*Very relevant presentation to general practice. Good discussion and learning from each other. Very good presentation of the data*.’ (Topic 2)

‘*Very relevant to general practice. Application of data and information change behaviour of prescribing, decision sharing. Using local data as a tool to apply today’s knowledge*.’ (Topic 3)

Based on the positive results of the pilot overall, an extension for a further six topics over the course of following year to allow the programme to be further developed and refined was agreed on and signed by the CCG and Pegasus Health.

## Discussion

### Summary

Results both from the ratings given and the comments provided by responders showed that the three-topic pilot had been positively received by attendees overall. The Small Group model was well liked, with the interactive discussion component singled out for favourable comment.

### Strengths and limitations

The resolution of initial technological issues and the stronger adaptation for the UK context as the pilot progressed demonstrated the bedding in of the model as the content developers, facilitators, and participants became more familiar with it. The high uptake of the Small Group model — with 68 of the 70 GPs in the CCG registering to attend, and the doubling in the number of attendees between the first and third topics — was a key strength of the pilot. Similarly, that 90% (61 of 68) enrolled GPs attended the third topic session showed that the new model was receiving significant support, easily equal to, or for the third topic exceeding, the average attendance in New Zealand. Another strength of the collaboration was the existing relationship between the CCG and the wider primary care system in Canterbury. There were individual GPs in the CCG who had been involved in the previous collaboration who took a leading role in promoting and advocating for the Small Group pilot among their colleagues. Reinforcing this was the presence in the Pegasus Health team of several members who had worked in both the UK and New Zealand, and so, understood how each health system operated. This prior experience was an important contributor to getting the Small Group programme up and running in such a short time (3 months) from first discussions in August 2021 to implementation in November.

The decline in the proportion of GPs who submitted an evaluation, however, must be considered a weakness. While nearly all the evaluations submitted scored the sessions highly, the proportion of evaluations received, which were only a little below the level of those in New Zealand for topic 1, declined to just over one-third for topic 2, and to less than one-third for topic 3. This was well short of the average of 55% returns that the Pegasus participants return or every topic. Exact reasons for non-response are known to be difficult to discover,^16^ though in-class evaluations appear to have a higher response rate than those that are done later or online, as happened here.^17^ The declining proportion also raises the question of whether those that were submitted were from the most enthusiastic GPs in the group, and therefore, more favourable towards the Small Group model than participants overall. The doubling of attendance during the pilot, however, can be set against this potential bias, suggesting that the CCG GPs, who were unaccustomed to being asked for feedback, had not been given sufficient explanation about the important contribution that their evaluations would provide for the future of the programme.

### Implications for practice

Based on the overall positive results of the three-topic pilot, it was agreed to extend the Small Group programme for a further six topics over the following year to allow it to be further tested and refined. The extension would provide an opportunity to actively promote the importance of submitting the evaluation form, and to address any negative feedback from the participants. Albeit coming from a small minority, negative feedback was a reminder that attention needed to be given to maximising participant engagement if the programme was to be sustainable. Extending the programme would allow a longer exposure for participants to the interactive Small Group model, which was a considerable culture change from the previous lecture-style format they had been used to.

The first pilot sessions showed that participants needed encouragement to interact and to be reminded that sharing their own experiences and learning from others’ was a core component of all group models of continuing professional education.^5,7,11,12^ The extension would also allow time to identify and measure behaviour change of GPs from the topics delivered in the first pilot. A follow-up of the data presented within the topics on pharmaceuticals use and referrals was planned 12 months subsequent to the meetings, to review change in clinician behaviours that the Small Group education model may have contributed to.

All of these issues are now being addressed as the second phase of the pilot progresses. Further developments are being planned, including the potential for further scaling up the implementation of the model to include groups of other primary care health professionals, as is done with Pegasus Health in Canterbury, New Zealand.

This intercontinental collaboration and three-topic pilot demonstrated that Pegasus Small Group education model could be transferred to primary care teams in a different health system outside of New Zealand within a short timeframe. The South Tyneside CCG was able to benefit from Pegasus Health’s 30 years of experience in selecting and developing interesting and relevant clinical topics for GPs, along with successfully adopting the Small Group model and mechanism for peer-to-peer delivery and support.

Using technology-enabled communication, training, materials development, and support demonstrated the potential for health systems to collaborate globally despite the geographical distance. Collaborating globally widens available opportunities, and potentially reduces duplication in effort and resources. A wider evaluation framework to assess the impact of these clinical quality education initiatives is needed in future phases, to determine the impact on patient care and articulate the benefits to the health system of supporting the small group peer education model.

## Data

The dataset relied on in this article is available from the corresponding author on reasonable request.
